# Protein Surface Matching by Combining Local and Global Geometric Information

**DOI:** 10.1371/journal.pone.0040540

**Published:** 2012-07-17

**Authors:** Leif Ellingson, Jinfeng Zhang

**Affiliations:** 1 Department of Mathematics and Statistics, Texas Tech University, Lubbock, Texas, United States of America; 2 Department of Statistics, Florida State University, Tallahassee, Florida, United States of America; King's College, London, United Kingdom

## Abstract

Comparison of the binding sites of proteins is an effective means for predicting protein functions based on their structure information. Despite the importance of this problem and much research in the past, it is still very challenging to predict the binding ligands from the atomic structures of protein binding sites. Here, we designed a new algorithm, TIPSA (Triangulation-based Iterative-closest-point for Protein Surface Alignment), based on the iterative closest point (ICP) algorithm. TIPSA aims to find the maximum number of atoms that can be superposed between two protein binding sites, where any pair of superposed atoms has a distance smaller than a given threshold. The search starts from similar tetrahedra between two binding sites obtained from 3D Delaunay triangulation and uses the Hungarian algorithm to find additional matched atoms. We found that, due to the plasticity of protein binding sites, matching the rigid body of point clouds of protein binding sites is not adequate for satisfactory binding ligand prediction. We further incorporated global geometric information, the radius of gyration of binding site atoms, and used nearest neighbor classification for binding site prediction. Tested on benchmark data, our method achieved a performance comparable to the best methods in the literature, while simultaneously providing the common atom set and atom correspondences.

## Introduction

The functions of individual proteins and genes are essential for understanding the functions of cells or organisms as a whole. Since proteins’ functions are determined by their structures, structural genomic (SG) projects have been initiated with the aim to solve representative proteins in each protein family [1], [2], [3]. The solved protein structures can be used to predict the structures of those homologous proteins, whose functions can then be deduced from their structures. At the same time, structural genomic projects have produced a large number of protein structures, whose functions are still unknown. As many as 26% of all SG structures deposited to PDB [4] are described as proteins of unknown function, or their functions are quite often referred to as putative [5]. Predicting the functions of proteins based on their structural information has become one of the major roadblocks towards the goal of well-annotated genomes.

Since proteins function by interacting with other molecules through binding sites (active sites), analysis of the binding site provides a direct means to infer the function of a protein. A common hypothesis is that proteins with similar functions should have binding sites with similar shape and chemical properties.

Many studies have been conducted based on the idea of comparing the putative binding site of a target protein with unknown function with the binding sites of proteins with known functions to infer the function of the target protein. These previous studies can be roughly divided into two classes: those using only structure information and those using both structure and sequence/evolutionary information. Among those using only structure information to match binding sites, they can be further divided into two classes: those based on point clouds [6], [7], [8], [9], [10] and those based on shapes of binding sites [11], [12], [13] or shape-based descriptors or features [14], [15], [16], [17], [18], [19].

Several algorithms have been developed for matching binding sites represented by point clouds. SPASM and RIGOR [20] scan a structural database for occurrences of structure motifs using a combinatorial search with constraints. Jess [21] matched structure templates based on constrained logic programming. Cavbase and eF-site used clique-based method [8], [22] to match structure templates formed by surface patches. SiteEngine [7], [23], SitesBase [24], [25], MultiBind [9] and TESS [6] applied geometric hashing algorithms [6], [7], [9], [10], [26], [27] to match protein surfaces and binding sites. In a study by Weskamp et al. [10], clique detection and geometric hashing are combined. IsoCleft [28] used a graph-matching-based method to detect 3D atomic similarities. In a very recent study, Dundas et al. have developed an order-independent surface alignment method based on a structure alignment algorithm designed by Chen et al. [29] and applied it to study metalloendopeptidase and NAD binding proteins [30]. The above methods produce the correspondences between the atoms/residues of two binding sites, which can be used to calculate similarity using rigid superposition, such as root-mean-square-deviation (RMSD).

Instead of matching binding sites represented by point clouds, other methods have extracted information related to the shapes of the binding sites, which is then used for binding site comparison. Hoffmann et al. introduced a similarity measure called sup-CK and utilized global information from binding sites to align the sites based on their principal axes [31]. This method calculates similarity using a Gaussian convolution kernel, which does not require correspondences between atoms of the two binding sites. Sael et al. developed 3D Zernike descriptors to characterize and compare protein surfaces [14], [18]. Xiong et al. used feature vectors based on distance of groups of atoms on binding sites [19]. Das et al. applied property-encoded shape distributions (PESD) constructed using both atom-atom distances and the properties of the atoms [32]. And Xie et al. developed a shape descriptor called the Geometric Potential that characterizes both local and global topological properties of the protein structures [33], [34].

While many of the approaches focus primarily on the structure of the binding site, sequence and evolutionary information have also been used in assisting structure-based function prediction. Binkowski et al. first identified possible binding sites from α-shape of protein surfaces [12], [13], which was then followed by a sequence alignment of binding site residues to detect the similarity of protein binding pockets [11], [35], [36], [37]. Lichtarge et al. developed the evolutionary trace (ET) method to identify important amino acids from multi-sequence alignments, which are then used to construct local structure templates to compare protein surfaces. [38], [39], [40], [41].

When similar local structure motifs or templates are identified, assessment of the statistical significance of the similarity also plays an important role in function inference. To avoid the drawback of RMSD as a measure based on rigid superposition, a modified RMSD, oRMSD was used to measure the similarity of local surface structures [35]. Other similarity measures such as the Tanimoto index (TI) [42], [43] and the Poisson index (PI) [44] have also been adopted in protein binding site comparison.

In a recent study by Kahraman and co-workers, it has been found that pockets binding the same ligand show greater variation in their shapes than can be accounted for by the conformational variability of the ligand [16]. They suggested that geometrical complementarity in general was not sufficient to drive molecular recognition. The data set created for this study has served as a benchmark for performance comparison [31].

In this paper, we have developed a method based on the iterative closest point (ICP) algorithm [45], [46] for superposing and comparing protein ligand binding sites using atom-level representation of protein surfaces. Compared to the original ICP algorithm, our algorithm starts from a multitude of initial local alignments derived from 3D Delaunay triangulations and uses the Hungarian algorithm to find additional matched atoms. This Triangulation-based Iterative-closest-point for Protein Surface Alignment (TIPSA) algorithm aims to find the maximum common atom set (MCAS), defined as the maximum number of superposable atoms between two binding sites where distance between any pair of matched atoms in the rigid superposition of the binding sites is smaller than a given threshold value. In addition to matched atoms, we incorporate other geometric information to further improve the accuracy in ligand classification. Our method was tested on the Kahraman [16] and Homogeneous [31] benchmark data with good performance. This paper builds upon a preliminary study [47] which was based on local geometric information only, and features more thorough analysis including additional, more detailed results, and improvements to the algorithm that reduce computational cost while improving classification performance.

## Methods

### Algorithm for Surface Matching

Our approach is to treat the atoms of a ligand binding site as a cloud of points with corresponding labels specifying the chemical properties of the atoms. We compare the binding sites represented by the point clouds to find maximum common atom sets. Many past studies have used this or similar criteria. The assumption is that if two binding sites share a significant number of superposable atoms, they may share similar functions, as well. Due to the complexity of the problem, one has to use heuristic methods to find sub-optimal solutions, as in the previous methods reviewed in the Introduction. In this study, we denote any subset of atoms that can be matched between two binding sites as a common atom set (CAS), and the largest of such sets as the maximum common atom set (MCAS). In addition to using the MCAS, we also explore other information from ligand binding sites that can be conveniently used to improve ligand binding site prediction.

A standard technique used for alignment and registration for point clouds is known as the Iterative Closest Point (ICP) algorithm, which was introduced by Chen and Medioni [46] and Besl and McKay [45]. ICP aligns and registers an unlabeled set of points *p* to a model set *X* by iteratively alternating between registration and alignment steps. Registration is obtained by finding the closest point *y* in *X* to each point *p_i_* in *p*, resulting in the corresponding set *Y*. An alignment is then obtained by finding the optimal rotation matrix *R* and translation vector *v* such that *p* is superposed onto *Y*. These two steps are repeated until the change in mean square error between *p* and *Y* falls beneath a desired threshold.

However, ICP cannot be directly applied for matching ligand binding sites for two reasons. Firstly, since the algorithm is deterministic, the results depend greatly on the initial alignment used and the algorithm may find only a local, non-global, minimum. Besl and McKay suggested solving this problem by considering a large number of initial rotation states while superposing the centers of mass of two objects. However, superposing the centers of two binding sites, in many cases, may not provide good initial matching. We also propose an alternative approach to solve this problem by instead aligning locally similar structures.

Secondly, ICP does not guarantee unique correspondence between atoms, as registration is performed one point at a time, not jointly. Additionally, this approach to registration does not utilize the labels on the points. For the purposes of this application, it is necessary to find unique correspondence and make use of the chemical labels. For the registration problem, we use the Hungarian algorithm to find the optimal correspondences between atoms in two binding sites given the rotation and translation matrices. During the alignment, we also take the atom types into account. A list of atom types is shown in Table 1, and we utilize the labels of atom subtypes provided in column 3 to restrict matches to those sharing more specific chemical properties.

**Table 1:** Atom types used in binding site matching.

*Atom Type*	*Atom Subtype*	*Label*
Carbon (C)	Carbonyl C	1
	Aliphatic C, CA, Other sp3 C,	2
	Aromatic C	3
Oxygen (O)	Backbone O and carbonyl O in Asn and Gln, carboxyl O in Asp and Glu	4
	Hydroxyl O in Ser, Thr and Tyr	5
Nitrogen (N)	Backbone N, TRP side chain NE1, GLN NE2, ASN ND2, ARG NE NE1 NE2, LYS NZ	6
	HIS side chain NE1, NE2	7
Hydrogen (H)	polar H	8
Sulfur (S)	Disulfide bond S, Met S, Cys S	2

To address the dependence of global alignment on the initial state, we propose to solve a local alignment problem first and build to a set of global alignment from which we can obtain improved solutions, rather than searching for the global solution immediately. The procedure of our method is described as follows:

#### 1. Delaunay triangulation

For each protein, we compute the 3-dimensional Delaunay triangulation to obtain a set of tetrahedra with labeled atoms as vertices. The two sets of tetrahedra are compared pair-wise in order to obtain similar pairs that act as seeds, which are used to obtain potential initial alignments for the matching process.

#### 2. Comparison of tetrahedra from two binding sites

These inter-protein tetrahedral pairs are first checked for identical chemical composition. For those pairs with matching chemical compositions, the structural similarity of the tetrahedra is checked using the Distance Root Mean Square Deviation (dRMSD), which can be calculated as follows:
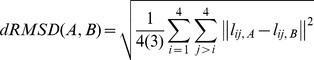
(1)where *l_ij,A_* is the length of the edge from atom *i* to atom *j* of the tetrahedron from the first protein and *l_ij,B_* is the length of the edge from atom *i* to atom *j* of the tetrahedron from the second protein. In order to simplify comparisons to the root mean square deviation (RMSD), we use the following alternative formulation that differs from (1) only by a multiplicative constant:



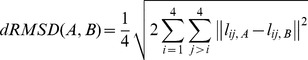
(2)This formulation can be used only because every tetrahedron has six edges, resulting in the two formulas differing only by the same constant for all pairs. At this stage, dRMSD is used in place of RMSD, which is utilized subsequently, in order to save on computational cost. The only pairs considered further are those with dRMSD values less than a 1.5 times a chosen RMSD cutoff value (1.25 Å). This cutoff for dRMSD was chosen based upon the relationship between pairs of RMSD values and corresponding dRMSD values for a large number of superpositions, for which we found that the dRMSD (2) for a superposition was no more than 1.5 times the associated RMSD value.

In many cases, the chemical composition for a tetrahedral pairing may lead to the possibility of multiple potential alignments. This occurs if there are multiple atoms of the same type within a tetrahedron. For example, if the tetrahedron consists of three carbon atoms and one oxygen atom, there are 6 possible alignments of the tetrahedra. In such instances, all possible alignments must be initially considered.

All of the tetrahedra in the two binding sites are compared and their dRMSD values are sorted. Ideally, all seed pairs satisfying this condition would be considered for alignment, but in many cases, doing so needlessly raises computational cost. Instead, we consider only those pairs with the lowest dRMSD values if the number of candidates is large. Using a benchmark data set, we tested a number of cutoff values to determine an appropriate number of seed pairs to be used (see Results) and arrived at using 500.

#### 3. Initial alignment

Once all pairs of tetrahedral seeds are obtained and sorted, the process of checking for additional matched atoms begins. For each seed pairing, one tetrahedron is held in a fixed position and the other is superposed onto it, yielding an optimal translation vector *v* that aligns the centers of mass for the seeds, and rotation matrix *R*, both of which are then applied to the moving protein, resulting in a rigid transformation that aligns the proteins at the location of the seed pairing. The translation vector is given by *v  =  B_C_ – A_C_,* where *A_C_* and *B_C_* are, respectively, the centers of mass for the coordinates *A* and *B*. The optimal rotation matrix *R* is calculated using the Kabsch algorithm [48], [49], which is an application to bioinformatics of the solution of the orthogonal Procrustes problem [50]. For this superposition, the RMSD is then calculated using the formula:
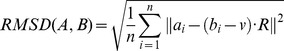
(3)where *a_i_* and *b_i_* are, respectively, the coordinates for the *i*th atom in *A* and *B*. If the RMSD for this configuration is less than the chosen cutoff value of 1.25 Å and det*R*  = 1, then additional matched atoms are searched for. Despite considering only tetrahedral pairs with low dRMSD at this point, it is still necessary to calculate the RMSD so as to solve the problem of multiple solutions discussed above, resulting in the removal of improper seed pairs. The restriction on the determinant of *R* is used to ensure that it is a true rotation matrix and not a rotation-reflection matrix.

#### 4. Atom Matching

Once the translation and rotation matrices are applied to the moving protein, we want to search for each atom of the moving protein an atom with the same type from the fixed protein with a distance smaller than a cutoff value, called the search radius (SR). To determine the matches for each atom, it does not suffice to consider each atom from the fixed protein separately due to the fact that doing so could lead to multiple fixed atoms sharing matches. For a given alignment, the solution to this matching problem, once the restrictions on labels and locality are imposed, is provided by the Hungarian algorithm [51], [52], which finds at most one unique match for each atom in the fixed protein, as implemented by [53]. To find the optimal search radius, we tested several reasonable values on a benchmark dataset (see Results).

#### 5. Iterative alignment

After the matched atoms are found for a pair of tetrahedral seeds, additional matched atoms are searched for by refining the optimal configuration of the proteins by expanding the seed to encompass all of the matched atoms for that superposition. To increase computational efficiency, those configurations that resulted in few atoms being matched are excluded from further consideration. For a given expanded seed, the optimal translation vector and rotation matrix are recalculated, as above. For this refined superposition, additional matched atoms are searched for. This process of refining and searching is repeated for a given seed pair until no additional matched atoms are found.

After using the iterative procedure to find the maximum number of matched atoms, the number of matched atoms is recorded. This process of superposing and searching for additional matches is repeated for all tetrahedral seed pairs beneath the chosen dRMSD cutoff. However, it can often be the case that multiple tetrahedral pairings will result in the same superposition of the moving protein onto the fixed protein. In order to avoid needlessly repeating the process in such cases, if the list of matched atoms includes all of the atoms from a remaining tetrahedral pair, then that pair is removed from consideration as a possible seed.

Upon completion of the above procedure, the optimal superposition is taken to be the configuration that results in the largest number of matched atoms. In the case that multiple configurations produce the largest number of matched atoms, the optimal configuration is taken to be that with the smallest RMSD. Accordingly, a list of the matched atoms is also obtained.

### Classification

To assess the performance of the algorithm, we perform classification of the ligands of protein binding sites using two benchmark data sets utilized by Hoffmann et al [31]. Since TIPSA aligns the binding sites by way of maximizing the CAS, a natural similarity measure to use is the Tanimoto Index (TI), which, in general, is defined as the ratio of the size of the intersection of two sets to the size of the union of those sets [54]. For our purposes, the TI is defined as follows:
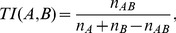
(4)where *n_A_* and *n_B_* are, respectively, the number of atoms in site A and the number of atoms in site B and *n_AB_* is the number of atoms common to sites A and B.

The primary measures of similarity considered by Hoffmann et al [31] do not utilize atom correspondence, but rather use a family of Gaussian convolution kernels to define similarity, which are referred to as sup-CK and sup-CK_L_. However, the authors also define the Sup-TI (referred to there as Sup-PI) measure, which is the result of finding matched atoms and calculating the TI following the completion of their correspondence-free alignment. Additionally, they consider Vol(A,B)  =  |Volume(A) – Volume(B)| as a measure of the difference in the sizes of the binding sites. Hoffmann et al [31] also consider some linear combinations of these measures.

Inspired by the idea that global geometric information of the binding site could bolster classification based solely on matched atoms, we consider a number of additional measures. RMSD is commonly used to measure structural similarity, but, since the CAS have varying numbers of atoms, it is desirable to normalize this measure. To do so, we utilize a version of the normalized RMSD of Carugo and Pongor (2001), calculated as.
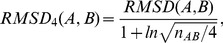
(5)which can be interpreted as being the RMSD value that would be observed for a pair of binding sites containing 4 atoms which exhibit the same amount of similarity as the binding sites that were actually compared. It is natural to use 4 atoms for the normalization since the initial step involves calculating the similarity of pairs of tetrahedra.

As shown in [31] and [16], the size of a binding site is useful for classification. While the TI only reduces the effect of the sizes of the binding sites for examining the number of matched atoms [44], an additional measure, such as Vol, is needed to include size information. The calculation of the volume of an active site is not trivial, though, so we consider an alternative method for incorporating size information that is easy to calculate, the radius of gyration *Rg*, which is calculated as follows:
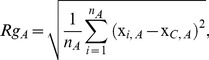
(6)where x*_i,A_* is the vector of coordinates of the *i*th atom from site A and x*_C,A_* is the vector of coordinates of the center of mass of site A. This provides a measure of the average distance between the atoms and center of mass of an active site. To utilize this information, we define the following similarity measure: *Gyr*(*A,B*) * =  |Rg_A_ - Rg_B_|*. The final measure that we examined provides information about the chemical composition of the binding sites. We consider the proportion of hydrophobic atoms present in the active sites, and define the measure *HydProp*(*A, B*) to be the square difference between the proportions for sites A and B.

In order to consider both local and global information, we explore linear combinations of these similarity measures. However, considerations must be made for doing so. First, increasing similarity between sites results in increasing values of TI, while this results in lower values for all other similarity measures. As such, it is necessary to utilize 1-TI instead of TI directly. Furthermore, since the measures are on different scales, in order to avoid one feature overly influencing the results, we divide all values of each similarity measure by the maximum observed value of that feature. Doing so allows the relative importance of each measure to be determined completely by the value of the weight placed upon it by the linear combination.

For the purposes of comparison to the above methods, performance is measured using classification error (CE), which is defined as the proportion of incorrect predictions. Using the scheme considered in the previous study, a classification is considered to be correct only if the predicted ligand exactly matches the actual binding ligand. The similarity of some of the ligands is not taken into consideration.

The results are obtained using the double leave-one-out cross validation method for *k*-nearest neighbor classification described in [31]. Using this procedure, classification is performed for a given binding site A by comparing it to all other sites except for some site B (the left out site). For site A, this process is repeated until each of the other sites has been left out. By doing so, it can be determined whether a particular classification is due to the presence of just one overly influential site. This entire process is then repeated for the classification of all sites in the data set.

### Data

The first set of data used for classification was compiled by Kahraman *et al.* [16]. The Kahraman data set consists of 100 active sites that are grouped according to which of 10 ligands they bind to. These ligands have varying amounts of flexibility. AMP, AND, EST, GLC, and PO4 are rigid; ATP, FMN, and HEM are moderately flexible; FAD and NAD are highly flexible. The ligands are also of varying sizes, with PO4 being the smallest and FAD being the largest. A summary of the binding sites included in the set is provided in Table 1 of [16].

The second data set we consider was compiled by Hoffmann et al [31] and will be referred to as the homogeneous data set. This set consists of 100 binding sites, with 10 groups of 10 binding sites each. However, the ligands these groups of sites bind to are all of similar sizes. The binding ligands for this set are PMP, SUC, LLP, LDA, BOG, PLM, SAM, U5P, GSH, and 1PE. A summary of the binding sites included in this set can be found in the online supplement of [31].

For the purposes of comparing classification results to those of previous studies, we initially define a binding site taken from a x-ray structure to consist of those atoms within 5.3 Å of the specified ligand [31]. To further explore the performance of TIPSA, we subsequently consider binding sites from these two data sets consisting of atoms within 7 Å of the binding ligands. For ease when discussing the data sets, we will subsequently only refer to this cutoff for the 7 Å sets.

### Optimization of the Search Radius and the Number of Nearest Neighbors

In order to select the optimal search radius for the algorithm, we performed all pairwise comparisons for the Kahraman data set for search radii of 1.0 Å, 1.5 Å, 2.0 Å, 2.5 Å and 3.0 Å using the double leave-one-out cross validation procedure. Table 2 shows the CE for these values of the search radius and for the nearest neighbor, 3-nearest neighbor, 4-nearest neighbor and 5-nearest neighbor classifiers. A 2-nearest neighbor classifier is not considered as it is guaranteed to produce identical results to the nearest neighbor.

**Table 2:** CE for studied combinations of search radius and classifiers.

*k-Nearest Neighbor*	*Search Radius*				
	1.0 Å	1.5 Å	2.0 Å	2.5 Å	3.0 Å
1	0.56	0.53	0.45	**0.43**	0.56
3	0.58	0.53	0.51	**0.42**	0.53
4	0.78	0.70	0.66	**0.66**	0.65
5	0.78	0.70	0.66	**0.66**	0.65

From Table 2, it is apparent that the optimal search radius is 2.5 Å. It appears that using a larger search radius defines similarity too loosely, resulting in dissimilar atoms being considered as matched. Using a smaller search radius appears to be too restrictive, not allowing for flexibility. The 3-nearest neighbor classifier performs marginally better compared to the nearest neighbor when using the TI alone, but not enough to rule out using *k* = 1. However, it appears that the 4- and 5-nearest neighbor classifiers perform substantially worse.

To more closely examine the optimal choice for *k*, we consider the linear combinations with the other similarity measures, as shown in Table 3. The CE are equal for *k* = 4 and *k* = 5. For the two linear combinations that perform better than TI alone, the nearest neighbor works better than the other classifiers. For RMSD_4_, the *k* = 3 and *k* = 5 perform marginally better than *k* = 1. Based upon these results, it appears that the nearest neighbor classifier (*k* = 1) provides the best results.

**Table 3:** CE for combinations of factors for various levels of k-nearest neighbor.

*k*	*1*	*3*	*4 and 5*
TI + Gyr	0.29	0.33	0.53
TI + RMSD_4_	0.43	0.41	0.66
TI + HydProp	0.36	0.40	0.64

## Results

### Ligand Classification Results

We first consider ligand classification using the Kahraman data set. A summary of CE for the methods discussed previously is provided in Table 4. Additionally, the table also presents CE for random classification. If there are no assumptions made about the relative frequency of each ligand class, then the CE is 0.90. However, if it is assumed that the relative frequency of each ligand class is known, then random classification results in an average CE of 0.87.

Our method with TI as a similarity measure, or TIPSA-TI, has a CE of 0.43. There is a negligible difference between this method and the previous methods Sup-TI and MultiBind, which both result in CE of 0.42, suggesting that TIPSA-TI compares well to these approaches when only a subset of matched atoms are considered in classification. While these CEs are far better than for random classification, performances achieved based on solely the common atom sets identified by these methods are still not very satisfactory compared to the Sup-CK (CE = 0.36) and Sup-CK_L_ (CE = 0.27) methods, which also use information from non-matched atoms.

The Vol measure alone performs fairly well, resulting in a CE of 0.39. However, using a linear combination of their Sup-CK and Sup-CK_L_ scores with Vol results in decreases in CE of, respectively, 0.02 and 0.01. The reason for such a small decrease is likely due to the fact that the Sup-CK and Sup-CK_L_ scores implicitly consider the sizes of the sites.

Table 4 shows that, while Gyr, HydProp, and RMSD_4_ all perform better than chance, each falls short of the previously discussed methods. The optimal linear combination, calculated by searching over a fine grid of weights, of each of these scores with TIPSA-TI shows some amount of improvement. HydProp improves classification slightly. The optimal linear combination places roughly 80% of the weight on HydProp. The CE for the optimal linear combination of RMSD_4_ and TIPSA-TI is 0.43, suggesting that this feature does not warrant being included.

**Table 4:** Results of *k*-nearest neighbor classification for the Kahraman (5.3 Å) data set.

Method	Classification Error
TIPSA-TI	0.43
TIPSA-TI + Gyr	**0.29**
TIPSA-TI + RMSD_4_	0.43
TIPSA-TI + HydProp	0.36
TIPSA-TI + Gyr + HydProp	**0.28**
Gyr	0.54
RMSD_4_	0.71
HydProp	0.64
Sup-CK	0.36
Sup-CK + Vol	0.34
Sup-CK_L_	**0.27**
Sup-CK_L_ + Vol	0.26
Vol	0.39
Sup-TI	0.42
MultiBind	0.42
Random (No Assumptions)	0.90
Random (Known Proportions)	0.87

Using a linear combination of Gyr with TIPSA-TI produces a CE of 0.29, which is comparable to Sup-CK_L_. However, our alignment method also identifies the important atoms through obtaining atom correspondences, while achieving the goal of classification at the same time. The optimal combination places 52% of the weight on Gyr, indicating both features contribute roughly the same amount towards the classification.

Since separate linear combinations with both Gyr and HydProp improved classification results, we considered utilizing all three features to perform classification. Using a linear combination of TIPSA-TI, Gyr, and HydProp with respective weights of 37.74%, 41.51% and 20.75% produces a CE of 0.28. This reduction in CE is marginal and does not necessarily warrant the inclusion of HydProp. However, further study shows that doing so may be useful, in general.

We now consider the Homogeneous data set. A summary of CE for this data can be found in Table 5. Using TI alone, we produce a CE of 0.49, which is comparable to the other correspondence based methods Sup-TI and MultiBind, which result in errors of, respectively, 0.47 and 0.48. However, Sup-CK_L_ and Sup-CK_L_ + Vol both have only 0.38 CE. As with the Kahraman set, the addition of Vol does not improve results.

**Table 5:** Results of *k*-nearest neighbor classification for the Homogeneous (5.3 Å) data set.

Method	Classification Error
TIPSA-TI	0.49
TIPSA-TI + Gyr	0.44
TIPSA-TI + RMSD_4_	0.49
TIPSA-TI + HydProp	0.42
TIPSA-TI + Gyr + HydProp	**0.38**
Gyr	0.77
RMSD_4_	0.58
HydProp	0.83
Sup-CK	0.47
Sup-CK + Vol	0.46
Sup-CK_L_	**0.38**
Sup-CK_L_ + Vol	**0.38**
Vol	0.89
Sup-TI	0.47
MultiBind	0.48
Random	0.90

Again, Gyr, RMSD_4_, and HydProp all perform better than random classification, but in this case, RMSD_4_ outperforms Gyr and HydProp considerably. Because the ligands in this set are all of similar size, it is sensible that Gyr would not be as useful here. Despite this, all three perform worse than TIPSA-TI.

The optimal combination of RMSD_4_ with TIPSA-TI places 100% of the weight on TIPSA-TI, suggesting, again, that this feature does not bring in useful information in addition to what is found by examining the number of matched atoms. The optimal combination of Gyr and TIPSA-TI places 49% weight on TI again and results in a CE of 0.44. Again, the optimal combination of TIPSA-TI and HydProp places 20% weight on TIPSA-TI, producing 0.42 CE. The optimal combination of TIPSA-TI, Gyr, and HydProp, which places weights of, respectively, 30%, 30%, and 40%, has a CE of just 0.38, which matches the performance of Sup-CK_L_.

For both data sets, the optimal weight placed on HydProp in linear combination with TIPSA-TI is higher than might be anticipated due to the poor performance when using it alone. However, this combination of similarity measures performs considerably better than HydProp alone for nearly all values of the weight, with most attaining a CE of no more than 0.50. With that said, the CE for this data is lower over the range of 50% to 85% for the weight of HydProp, so any choice of weight in this range would produce similar results.

### Effect of the Hungarian Algorithm and Iterative Alignment on Classification

While there is certainly conceptual justification for the use of the Hungarian algorithm to perform the matching for a given alignment, we also wanted to examine the effect of this procedure on ligand classification. Additionally, we also considered the impact of iterative alignment. To test these, we removed each aspect separately and jointly from the algorithm. Classification errors for the Kahraman set using these altered versions for optimal linear combinations of TI and Gyr as similarity measures are provided in Table 6.

**Table 6:** CE for the Kahraman (5.3 Å) data set from alternate versions using TI + Gyr to measure similarity.

Method	Classification Error
Standard	**0.29**
No Hungarian	0.33
No Iterative Alignment	0.30
No Hungarian and Iterative Alignment	0.32

The difference in CE between the standard implementation of TIPSA and that without iterative alignment is negligible, but the increase in computational cost is also negligible. In addition, while the classification results do not significantly improve with the iterative alignment, this aspect is still vital for arriving at the optimal alignments.

However, there is a more substantial increase in CE when the Hungarian algorithm is removed from the procedure. This further supports the notion that it is important to obtain atom correspondences simultaneously rather than consecutively.

The implementation without either aspect of the algorithm also shows an increase in CE from the standard implementation. Since this particular version is without two of the key distinguishing features of TIPSA, it is the implementation that is the most similar of these examined to other methods, though there are still key differences. For example, while much of this implementation is similar to the methods employed by SitesBase, the seed pairs there are determined using triangles rather than tetrahedra. Despite such difference, these results may help provide some insight into the differences in performance between TIPSA and other methods, which could be useful when considering those methods with which we cannot directly compare. Most importantly, however, we can see that the combination of the Hungarian algorithm and iterative alignment improves performance.

### Binding site Classification Compared to Ligand Similarity

Recall that in the scheme used, a classification was only considered correct if the binding ligands were identical; ligand similarity was not taken into account. Selecting the TIPSA-TI + Gyr model as optimal, we explored the erroneous classifications for the Kahraman set to gain a better understanding of the results. Using the cross validation procedure, the 29 binding sites listed in Table 7 were all classified correctly at most once while all of the other binding sites were *misclassified* at most once, meaning that these 29 sites account for nearly all classification errors.

**Table 7:** The misclassified binding sites (classified ligands in parentheses).

*Ligand*	*PDB IDs for the Misclassified Active Sites*
AMP	12as (ATP), 1 amu (EST), 1c0a (GLC), 1 jp4 (ATP), 1 kht (EST), 1 tb7 (AND), **8 gpb (PO4)**
ATP	1b8a (AMP), 1 dy3 (FMN), 1 esq (AMP), 1 gn8 (FMN), **1o9t (NAD)**, 1 tid (EST)
FAD	**1jr8 (HEM)**
FMN	1f5v (ATP), 1ja1 (EST), **1 mvl (GLC)**, **1 p4m (NAD)**
GLC	*None*
HEM	**1qpa (NAD)**
NAD	**1ej2 (PO4)**, **1ib0 (AMP)**, **1o04 (HEM)**, **1tox (AND)**
PO4	1e9g (GLC), **1gyp (ATP)**, **1l7m (NAD)**
AND	1e3r (EST), **1j99 (NAD)**
EST	1fds (AND)

**Figure 1:**
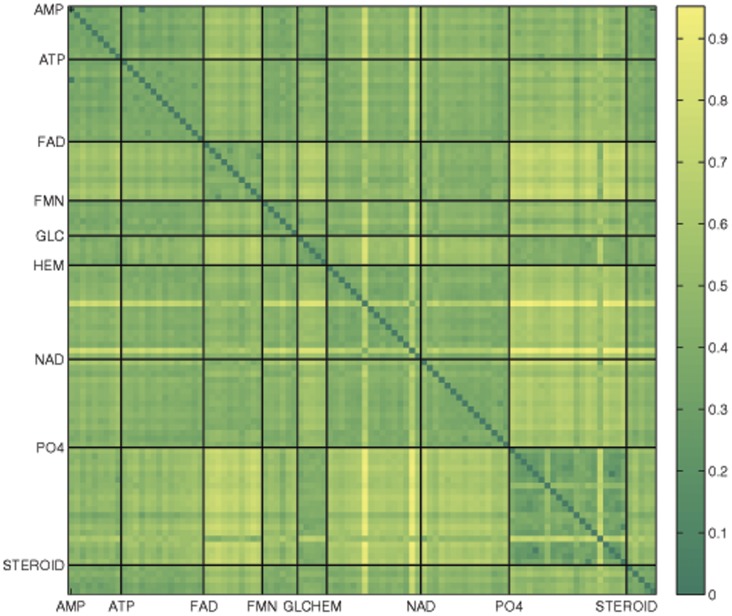
Similarity matrix computed using the TIPSA-TI + Gyr model. Dark pixels represent greater similarity and light pixels correspond to less similarity. The steroid group consists of both the AND and EST ligand groups in accordance to Kahraman *et al* (2007).

The similarity matrix for the TIPSA-TI + Gyr model is shown in Fig. 1. The ligand groups that produced the highest proportion of missed classifications are AMP, ATP, FMN, EST, and AND. From Fig. 1, we see that the AMP, ATP, and FMN sites are troublesome due to the high amount of similarity with other ligand groups. The EST and AND sites are misclassified largely due to the small number of sites binding to these ligands in the data. For the purposes of comparing results with Hoffmann *et al* (2010), these two ligands were considered separately. However, as presented in Kahraman *et al* (2007), these two groups can be combined and thought of as a steroid group, as displayed in Fig. 1.

Kahraman *et al* (2007) examined the structural similarity of the ligands of these binding sites using spherical harmonic coefficients. The ligand similarity matrix obtained in that study has a similar overall pattern to what is shown in Fig. 1, suggesting that our results for the similarity of the binding sites are largely consistent with the underlying similarities of the ligands themselves. Among the 29 misclassified binding site pairs (Table 7), 16 of them bind to ligands that are structurally similar to those they were identified as. This brings unexplained classification error to 0.13.

Unfortunately, we are not able to compare our unexplained error to other methods because similar results have not been provided. Nonetheless, it is important to remember that, while perfect classification would be ideal, it is perhaps more reasonable to expect correct classification of binding ligand only up to limitations due to structural similarities of the ligands.

### Approximation of Binding Pockets

While validation of the methodology requires using sites that are known to bind to a given ligand, this is not ultimately of so much practical interest. Instead, a problem of greater interest, as well as challenge, is to predict the binding ligand without knowing the location of the binding site on a protein surfaceconsider pockets on the surface of the protein that are of unknown function. However, since classification error for this problem is confounded with binding site prediction error, any results obtained would be contingent upon the method used for binding site prediction. As such, in this study, rather than directly considering this problem, we approximate this scenario by utilizing larger binding sites, defined to consist of all atoms within 7 Å of the bound ligand.

Table 8 presents classification results for both benchmark data sets in this more challenging scenario. Performance on both data sets has decreased somewhat, but this change in performance is more pronounced for the Kahraman (7 Å) set. Perhaps of the most interest, though, is that RMSD_4_ appears to be more important for classification with these larger sites, as linear combinations with TIPSA-TI reduce error considerably. For the Homogeneous (7 Å) data set, the linear combination including all four features produces the best results, with 20% TIPSA-TI, 10% Gyr, 60% HydProp, and 10% RMSD_4_. The optimal combination for the Kahraman set consists of 10% TIPSA-TI, 30% Gyr, and 60% HydProp.

**Table 8:** Results of *k*-nearest neighbor classification for the Kahraman and Homogeneous data sets.

Method	Classification Error	
	Kahraman	Homogeneous
TIPSA-TI	0.53	0.52
TIPSA-TI + Gyr	0.41	0.49
TIPSA-TI + RMSD_4_	0.38	0.46
TIPSA-TI + HydProp	0.38	0.48
TIPSA-TI + Gyr + HydProp	**0.36**	0.45
TIPSA-TI + Gyr + HydProp + RMSD_4_	**0.36**	**0.42**
Gyr	0.55	0.84
RMSD_4_	0.70	0.52
HydProp	0.80	0.81

Binding sites consist of all atoms within 7 Å of the ligand.

The changes in the weights and the increase in CE are likely due to the additional atoms now included in the sites. Because the additional atoms are all, by definition, further from the centers of mass of the binding sites, they may have a greater influence on the alignments than those atoms that are closer to the centers of mass. As such, it would be of interest in the future to study this more closely to gain a better understanding of the problem and to find a way to adjust for it accordingly.

### Reduction in Number of Seed Pairs Considered

If computational speed were not a factor, TIPSA could ideally be implemented using all seed pairs satisfying the similarity constraints. However, for some pairs of binding sites, this number could be prohibitively large. As such, it is important to determine a rough lower bound for the number of seed pairs considered for alignment, so as to retain accuracy while also keeping computational cost at a minimum.

In order to explore this problem, we performed classification for the Kahraman set using various numbers of seed pairs, recording classification error and average runtime per alignment for each. These summaries are displayed in Table 9. For timing purposes, all computations were performed using MATLAB on a machine running Windows 7 on an Intel Quad-Core Xeon processor running at 2.4 GHz.

**Table 9:** CE and average runtime per alignment for various seed pair restrictions.

Number of Seed Pairs	Classification Error	Average Runtime (sec)
1000	0.29	11.4
500	0.29	6.1
450	0.33	4.7
400	0.32	4.1
300	0.27	2.3
100	0.33	1.2

It appears that none of the seed pairs beyond the best 500 provide any benefit for alignment, so TIPSA should be run with at most 500 seed pairs under consideration. Doing so cuts the computational cost roughly in half.

However, further reducing the number of seed pairs used results in instability in the classification results. For example, while using 300 seed pairs further decreases the computational cost to an average of just 2.3 seconds per alignment, we also see an increase in classification error. The incorrect classifications are nearly identical as those shown previously. However, 5 of the previously misclassified sites are now classified correctly, with 3 of those being ones that were considered unexplained by ligand shape, while 3 other sites are now misclassified, two of which are not immediately accounted for by similarity in ligand shape. This brings the unexplained classification error to 0.12.

Despite this reduction in classification error, the fact that previously correct classifications are now misclassified shows that a few good alignments are thrown away here along with some “bad,” which were most likely found due to coincidental alignments. This, along with a subsequent raise in CE for similar numbers of seed pairs (Table 9), suggests that we should only reduce the number of pairs considered up to the point at which the results of the algorithm are not affected.

### Computational Considerations

The running time of TIPSA varies depending on the number of atoms in each binding site and the similarity of the binding sites, as well. In general, the runtime is shorter for smaller binding sites. Furthermore, as discussed previously, the running time of TIPSA depends on the number of seed pairs used. Keeping these factors in mind, TIPSA requires an average of between 2 and 6 seconds per binding site pair to perform alignment. These runtimes were obtained by implementing TIPSA using MATLAB on a machine running Windows 7 with an Intel Quad-Core Xeon processor running at 2.4 GHz. In order to increase the computational efficiency of TIPSA, one goal is to develop an alternative implementation of the algorithm in a compiled programming language, such as C++, rather than MATLAB. This should cut down on computational costs considerably, as MATLAB, being an interpreted language, is not so conducive to nested series of large loops. However, even without such an adaptation of the code, the algorithm can be run in parallel, further cutting down on runtime for pairwise comparisons. The reported running time for sup-CK ranges from 0.2 and 1.3 seconds per comparison when running on a machine with a 2.5 GHz CPU. While timing results for MultiBind were not reported for these data sets, [9] reports run times ranging between 8 and 58 minutes for *multiple* alignments of groups of 5 binding sites when utilizing an Intel Pentium IV 2.60 GHz processor. It should be noted that MultiBind’s computational time varies for similar reasons as TIPSA.

### Identification of Maximum Common Atom set (MCAS) from Pairs of Binding Sites

We verify whether TIPSA can successfully find good solutions for the common atom set (CAS) from two binding sites. Although a proof that the obtained solution is, indeed, the MCAS is difficult to provide, the algorithm is designed so that, subject to a chosen search radius, the number of matched atoms is maximized for a given initial alignment of tetrahedral seeds by utilizing the Hungarian algorithm and iterative realignment. As such, the CAS found by TIPSA will be maximal for the set of initial alignments considered, subject to the locality restrictions imposed. In order to illustrate this, we have compared the matching results of TIPSA with SitesBase, which is based on geometric hashing. While a large-scale comparison to SitesBase is not currently possible because the programs are not readily available, we present two detailed examples comparing our algorithm to SitesBase.

To illustrate how the differences in methodology can impact the resulting set of matched atoms, we first consider the following example. The top ranked match on SitesBase for the AMP binding site of protein 1ct9 that is not another site from 1ct9 is the APC site of protein 1q19. For the purposes of this example, we utilize the definition of an active site used for SitesBase; the sites consist of all atoms within 5 Å of the ligand molecule. Table 10 displays the atom correspondences for atoms in the query site that are matched differently by SitesBase and TIPSA.

**Table 10:** Matched atoms from the AMP binding site of 1ct9 and APC binding site of 1q19 found only by either SitesBase or TIPSA.

	1ct9 AMP	1q19 APC SitesBase	1q19 APC TIPSA
2	CA 232	C 244	CA 244
3	C 232		C 244
6	CG 232	CG 244	CD 244
7	CD1 232		CG 244
19	CB 238		CB 250
20	CG 238		CG 250
21	OD1 238		OD2 250
25	OG 239		OG 251
31	O 271		O 269
39	CB 279		CB 277
40	CG 279		CG 277
41	OD1 279	OE1 277	
42	OD2 279		OE1 277
43	SD 329		CG 327
44	SD 332		CG2 330
47	N 346	N 343	
48	CA 346	CB 343	CA 343
50	O 346		O 343
51	CB 346	CG2 343	CB 343
59	CB 348	CG 345	CB 345
60	CG 348		CG 345
71	NZ 449		N 444

**Figure 2:**
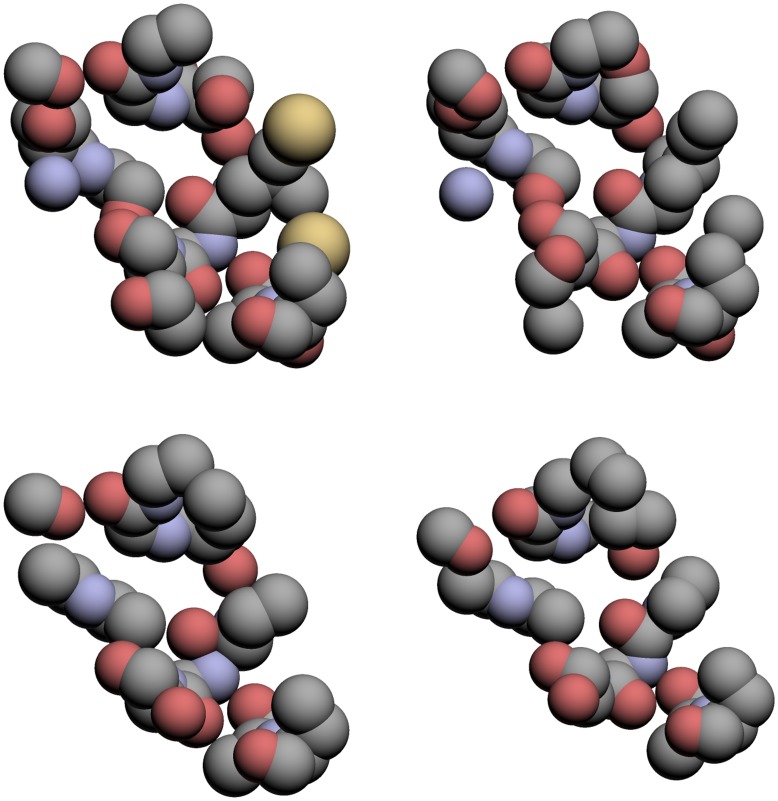
The atoms common to the ATP binding site of 1ct9 and the APC binding site of 1q19 as found using TIPSA (top) and SitesBase (bottom).

SitesBase found 46 atoms in common between these two sites, whereas TIPSA found 59 (Table 10, Fig. 2). A number of these additional matches can be attributed to a shift in the correspondences. For example, while atom C of residue 244 (C 244) from 1q19 is identified as a match using both algorithms, the atom it is matched to differs. SitesBase matches it to atom CA of residue 232 (CA 232) of 1ct9, whereas our method aligns it to atom C 232 and matches atom CA 244 to atom CA 232. This difference is likely due to the use of the Hungarian algorithm since it solves the correspondence problem for all atoms simultaneously. Other differences are likely due to the iterative alignment process of TIPSA. We found three atoms on residue 238 of 1ct9, which matched with three atoms on residue 250 of 1q19, indicating that this is a meaningful match. SitesBase missed atoms on these two residue altogether. Similarly, we matched three atoms on residue 279 of 1ct9 with three atoms on residue 277 of 1q19, while SitesBase only found one pair of match atoms. Not only can TIPSA obtain more matched atoms, it also does better in terms of matching atoms of the same type.

**Figure 3:**
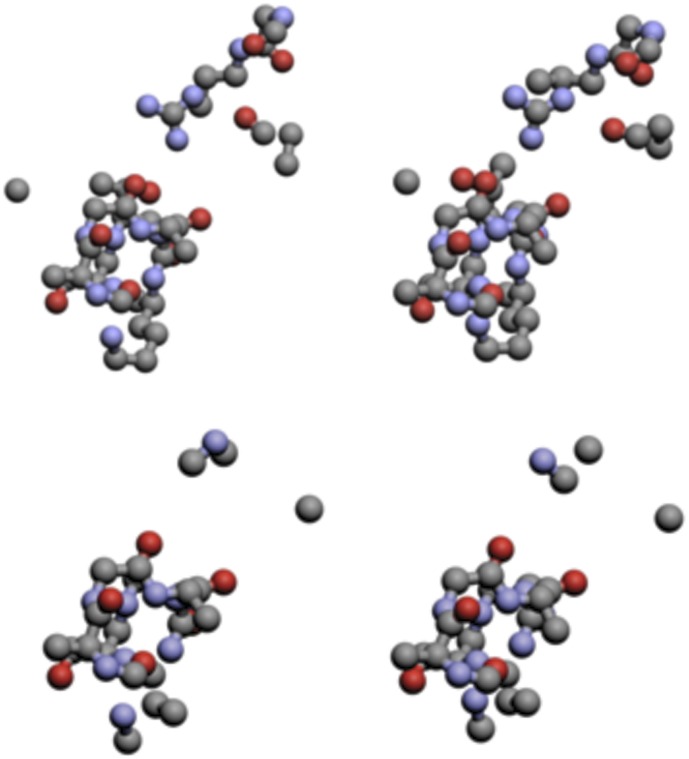
The atoms common to the ATP binding sites of, respectively, 1ayl and 1e2q as found using TIPSA (top) and SitesBase (bottom).

To further show how the maximum common atom sets from TIPSA compare to those of SitesBase, we consider the ATP binding sites of 1ayl and 1e2q, which are found in both the Kahraman set and the SitesBase set. SitesBase found a common atom set of size 38, whereas TIPSA found a common atom set of size 59. The methods agreed on all but 25 atoms. We found 23 matched atoms in the ATP binding site of 1e2q that SitesBase did not, while it found only 1 matched atom that we did not. These differences can be accounted for similarly to the previous case. Table 11 displays those atom correspondences for which the methods did not agree. The matched atoms found using TIPSA and SitesBase are displayed in Fig. 3, which shows that, while both methods obtain similar CAS, our algorithm is able to find the additional common atoms displayed in the upper portion of Fig. 3.

**Table 11:** Matched atoms from the ATP binding site of 1ayl and ATP binding site of 1e2q found only by either SitesBase or TIPSA.

	1ayl.ATP	1e2q.ATPSitesBase	1e2q.ATPTIPSA
37	CA 254		CA 19
41	CD 254		CD 19
58	C 256		C 21
59	CB 256		CG2 21
60	OG1 256		OG1 21
61	CG2 256		CB 21
78	CE 288		CG 16
98	CA 441	C 180	
99	C 441		C 180
100	O 441		O 180
108	NE 449	NE 143	NH2 143
110	NH1 449		NE 143
111	NH2 449		NH1 143
119	C 450		C 182
120	O 182		O 182
126	N 451		N 183
127	CA 451		CA 183
128	C 451		C 183
129	O 451		O 183
131	N 452		N 184
132	CA 452		CA 184
135	CB 452		CB 184
136	CG1 452		CG1 184
139	CB 455		CG2 187
142	CG2 455		CB 187

For both of these examples, the binding ligands are either identical or are structurally similar. For such cases, it should be expected that alignment methods would find very similar CAS if they are indeed finding common structures. Despite the differences in CAS, TIPSA found similar sets of common atoms to SitesBase for both pairs, but was able to identify additional matches, suggesting that our approach is able to find more atoms in the MCAS for similar binding sites. In performing these comparisons, we used a search radius of 2.5 Å for reasons described in a following section. This puts an upper bound for the resulting RMSD calculated from the obtained CAS at 2.5 Å.

## Discussion

In this study, we developed the TIPSA algorithm for comparison of protein binding sites based on the iterative closest point (ICP) algorithm originally designed in computer vision for matching objects represented by point clouds. We addressed the starting-point problem using similar tetrahedra from two binding sites that need to be compared, which allows us to efficiently find good solutions. We applied the Hungarian algorithm in finding the optimal matched atoms at each iteration step in ICP and found it significantly improved the matching results. To classify the binding sites according to binding ligand, we further incorporate global geometric information in the form of the radius of gyration of a binding site, and achieved a performance comparable to the best performance in previous studies. While the previous best performing method [31] does not obtain the common atom set between binding sites and the correspondences among the atoms, TIPSA does. The common atom set and atom correspondences will permit future analysis to characterize unique patterns occurring in a group of binding sites bind to the same ligand and detailed studies of structure-function relationships. The linear combination of scores from the factors we investigated allows a greater understanding of the roles the factors play in classification.

While TIPSA is similar in general to ICP, the aspects that differ allow us to better address the issues that arise in the problem of matching protein binding sites. To solve the dependence on the initial alignment state, Besl and McKay (1992) suggest using a dense set of rotations to initialize the procedure. This approach works well if there is no additional information available to narrow down the initial alignments. However, by utilizing the tetrahedra from Delaunay triangulation to determine initial alignments, we are able to make use of the data, itself, to focus the alignment search to include only those that have the most promising outcomes.

By incorporating atom labels, we are able to further reduce the number of initial alignments to only those that are chemically feasible. The labels also greatly aid in the matching of atoms by considering correspondences only between atoms of the same type. This aids in the alignment process by using only these atoms of interest in the calculation of the alignment operators. Without using the labels, ICP considers all of the atoms from the moving binding site in the alignment step. This is problematic since the non-matched atoms can be thought of as noise that obscures the alignment.

By utilizing the Hungarian algorithm, TIPSA is able to determine the optimal set of *unique* correspondences for a given superposition of two binding sites. Furthermore, we restrict the set of potential matches to consist only of those atoms from the moving protein that are near to the query atom from the stationary protein. This censoring prevents an inflation of the number of matched pairs by ensuring that matches are local. The method for obtaining correspondences using original ICP, while avoiding the locality problem, obtains a correspondence for every atom in the fixed binding site. This fails to solve the primary problem of identifying the common atom set. Another advantage of using a search radius is that the upper bound on the RMSD calculated from the matched atoms of two binding sites will be equal to the search radius.

The geometric hashing algorithm employed in the construction of SitesBase and other methods (see Introduction for references) has some similarity to our algorithm. However, there are a number of key distinctions. First, we use the Hungarian algorithm to find the set of one-to-one atom correspondences, which is optimal given a particular superposition. In most of the methods using geometric hashing, the optimal set of correspondences is not necessarily found. Secondly, the alignment process is not iterative in geometric hashing. While this maintains the optimal alignment for the triplet pairs, it does not account for the information provided by the remaining atoms, including those that are determined to be matching.

The MultiBind method also employs a form of geometric hashing, Unfortunately, we were not able to compare MultiBind in terms of its ability of finding CAS because their code is not readily available and the results in the online database were computed offline. As a result, any comparisons to SitesBase have to be made on an individual basis, so we were only able to directly compare on a few selected examples.

With CAS identified between similar binding sites, one can use multiple similar binding sites to define 3D patterns for each ligand and use the 3D pattern for ligand prediction. This will further speed up the classification process and may achieve even better accuracy. Since our method can better identify the common atom set, it may be more advantageous for characterizing common 3D patterns of binding sites in future studies.

We found that using the common atom sets alone is not adequate to predict the ligands of binding sites. Other information needs to be incorporated to account for the flexibility of ligands. No matter what conformation a ligand takes, the overall size of the pocket that binds the ligand does not change much, which is why the radius of gyration and volume of the binding sites or pockets play a role in classification. Our method uses radius of gyration as the global feature to predict the ligands of protein binding sites. Compared to the volume used in Hoffmann et al (2010), the radius of gyration offer two advantages. Firstly, it is very convenient and efficient to compute radius of gyration for any set of atoms. Secondly, some binding sites may be relatively flat, such as protein-protein binding sites, for which the binding site volume can be difficult to define. In such cases, radius of gyration should still provide the information on the sizes of the binding sites.

In this study, we extract binding site atoms using the atom coordinates of ligands in the same manner as previous methods. This insures that our results are comparable to previous studies. However, in a practical ligand prediction situation, the ligand information is usually not available. The binding sites often need to be predicted. In this study, we decouple the problem of binding site prediction from binding ligand prediction by assuming a perfect binding site prediction. It is expected that if binding sites are predicted instead of extracted using ligand information, the overall prediction accuracy would decrease. Najmanovich *et al.* [28] have investigated the effect of different ways of obtaining binding sites on the prediction performance.

If we were to use such predicted binding sites in this study, classification error would be confounded with binding site prediction error, making accuracy of the methodology presented here substantially more difficult to judge. However, to simulate predicted binding pockets, we also performed the classification procedure for the Kahraman and Homogeneous sets, but with the sites consisting of all atoms within 7 Å of the binding ligand. The classification errors for these version of the data were, respectively, 0.36 and 0.42. This sort of decrease in accuracy is to be expected as the additional atoms can obscure the patterns present in the atoms closest to the ligands.

In this study, we have not considered the flexibility or conformational heterogeneity in binding site prediction. Due in part to the flexibility of a number of the ligands and binding sites, it is possible that our algorithm finds only a local alignment for a given pair of binding sites. For such pairs, there may be multiple regions of similarity that cannot all be captured by the same alignment. To account for these cases, it may be advantageous to consider multiple alignments beyond that which produced the largest CAS. This may help to achieve even better classification results by alleviating partially the problem of protein/ligand flexibility, and to better understand the relationship between the structure and function of a binding site.
